# Lipopolysaccharide Induces Degradation of Connexin43 in Rat Astrocytes via the Ubiquitin-Proteasome Proteolytic Pathway

**DOI:** 10.1371/journal.pone.0079350

**Published:** 2013-11-13

**Authors:** Chih-Kai Liao, Chung-Jiuan Jeng, Hwai-Shi Wang, Shu-Huei Wang, Jiahn-Chun Wu

**Affiliations:** 1 Department of Anatomy and Cell Biology, College of Medicine, National Taiwan University, Taipei, Taiwan; 2 Institute of Anatomy and Cell Biology, School of Medicine, National Yang-Ming University, Taipei, Taiwan; Hertie Institute for Clinical Brain Research and German Center for Neurodegenerative Diseases, Germany

## Abstract

The astrocytic syncytium plays a critical role in maintaining the homeostasis of the brain through the regulation of gap junction intercellular communication (GJIC). Changes to GJIC in response to inflammatory stimuli in astrocytes may have serious effects on the brain. We have previously shown that lipopolysaccharide (LPS) reduces connexin43 (Cx43) expression and GJIC in cultured rat astrocytes via a toll-like receptor 4-mediated signaling pathway. In the present study, treatment of astrocytes with LPS resulted in a significant increase in levels of the phosphorylated forms of stress-activated protein kinase/c-Jun N-terminal kinase (SAPK/JNK) -1, -2, and -3 for up to 18 h. An increase in nuclear transcription factor NF-κB levels was also observed after 8 h of LPS treatment and was sustained for up to 18 h. The LPS-induced decrease in Cx43 protein levels and inhibition of GJIC were blocked by the SAPK/JNK inhibitor SP600125, but not by the NF-κB inhibitor BAY11-7082. Following blockade of de novo protein synthesis by cycloheximide, LPS accelerated Cx43 degradation**.** Moreover, the LPS-induced downregulation of Cx43 was blocked following inhibition of 26S proteasome activity using the reversible proteasome inhibitor MG132 or the irreversible proteasome inhibitor lactacystin. Immunoprecipitation analyses revealed an increased association of Cx43 with both ubiquitin and E3 ubiquitin ligase Nedd4 in astrocytes after LPS stimulation for 6 h and this effect was prevented by SP600125. Taken together, these results suggest that LPS stimulation leads to downregulation of Cx43 expression and GJIC in rat astrocytes by activation of SAPK/JNK and the ubiquitin-proteasome proteolytic pathway.

## Introduction

Astrocytes are the predominant supportive glial cells in the brain, where they participate in the formation of the blood-brain barrier and contribute to the maintenance of homeostasis in the central nervous system (CNS). Astroglial networks are interconnected through clusters of intercellular channels named gap junctions, which allow diffusion of second messengers, ions, and small metabolites between adjacent astrocytes [1]. Each gap junction channel is generated by the docking of two end-to-end hemichannels, termed connexons, in the opposing plasma membranes [2]. Connexon is composed of six membrane-spanning proteins named connexins (Cxs). Despite several Cx members that have been detected in astrocytes in many brain regions and in the hippocampus such as Cx43, Cx30, and Cx26, the major gap junction protein present in astrocytes *in vitro* is Cx43 [3], [4]. Evidence indicates that pathological and inflammatory stimuli affect Cx43 mRNA and protein levels and its phosphorylation state in astrocytes and thereby regulate gap junction intercellular communication (GJIC) [5]. Deletion of astrocyte Cx43 and Cx30 in double knock-out mice leads to myelin pathology, hippocampal vacuolation, and functional impairments in sensorimotor and spatial memory [6], [7].

The innate immune response in the CNS, triggered by activation of toll-like receptors (TLRs), is involved in response to non-infectious and infectious diseases, such as Parkinson’s disease, Alzheimer’s disease, multiple sclerosis, stroke, and bacterial meningitis [8], [9]. Stimulation of CNS-resident astrocytes with TLR agonists leads them to display several TLRs, such as TLR2, TLR3, and TLR4, which then bind adaptor proteins, i.e. a myeloid differentiation factor 88 (MyD88) and/or a TIR-containing adaptor molecule, Toll/interferon-1 receptor domain-containing adaptor inducing interferon-β (TRIF) [10]. Both the MyD88- and TRIF-dependent pathways induce the production of nitric oxide and proinflammatory factors via activation of p38, extracellular signal-regulated kinase (ERK), c-Jun N-terminal kinase/stress-activated protein kinase stress (JNK/SAPK), and nuclear factor-κB (NF-κB) signaling [9], [11]. Recent studies have shown that TLR activation in astrocytes results in reduced Cx43 expression and GJIC inhibition. For example, these effects are caused by treatment with polyinosinic-cytidylic acid (poly I:C), a TLR3 agonist [12], by TLR2 activation by the Gram-positive bacteria cell wall component peptidoglycan (PGN) or *Staphylococcus aureus* [13], and by exposure of astrocytes to lipopolysaccharide (LPS), a cell wall component of Gram-negative bacteria, via the TLR4 signaling pathway [14].

It has been suggested that ubiquitination plays a regulatory role in Cx43 internalization and endocytic trafficking [15]. Ubiquitination is a process that involves the covalent modification of the target protein by ubiquitin, a small protein consisting of 76 conserved amino acids [16]. Attachment of ubiquitin is mediated by a sequential cascade involving three classes of enzymes: E1 (a ubiquitin-activating enzymes), E2 (a ubiquitin-conjugating enzyme), and E3 (a ubiquitin-protein ligase) [16]. A previous study using specific pharmacological inhibitors demonstrated the participation of both the proteasomal and lysosomal pathways in Cx43 degradation [17]. The association of ubiquitin with Cx43 has been implicated as a pivotal step in the proteasomal and lysosomal proteolysis of Cx43 [15], [18].

During inflammation, tumor necrotic factor (TNF)-α-induced degradation of Cx43 and inhibition of GJIC in corneal fibroblasts is mediated by the ubiquitin-proteasome pathway [19]. A recent study reported that classical swine fever virus reduces endothelial Cx43 expression and GJIC via activation of the ERK, JNK, lysosome, and proteasome signaling pathways [20]. However, the mechanism targeting Cx43 for degradation in LPS-stimulated astrocytes remains poorly understood. In the present study, we investigated the possible signaling pathways involved in the LPS-induced degradation of Cx43 in cultured rat astrocytes. Our results provide evidence that the ubiquitin-proteasome proteolytic pathway plays a crucial role in regulating Cx43 expression and GJIC in astrocytes during LPS-mediated inflammation.

## Materials and Methods

### Ethics Statement

Animal protocols were approved by the Institutional Animal Care and Use Committee of National Yang-Ming University (IACUC permit number: 1011218). Humane care for all animals was observed, in compliance with the Guide for the Care and Use of Laboratory Animals as adopted and promulgated by the United States National Institutes of Health (NIH publication No.85-23, revised 1985).

### Primary Culture of Rat Astrocytes

Two-day-old Sprague-Dawley rats were purchased from the Laboratory Animal Center of the National Yang-Ming University and were euthanized by decapitation. Primary astrocyte cultures were prepared from the cerebral cortexes of rats as described previously [14]. Astrocytes were cultured on rat tail collagen-coated 35 mm culture dishes with glass coverslips at a density of 3.2×10^4^ cells per cm^2^ or without glass coverslips at a density of 6.5×10^4^ cells per cm^2^. Following incubation for 3 days, the enriched primary astrocytes were used for experiments. Confluent primary astrocytes were immunostained for glial fibrillary acidic protein (GFAP) and counterstained with DAPI for nuclei. Images were acquired using a 10X objective and the purity of primary astrocyte cultures was determined in a broad field of view. The population of GFAP-positive and DAPI-stained astrocytes was over 95% (Fig. S1).

### Reagents and Antibodies

Nitrobluetetrazolium (NBT), 5-bromo-4-chloro-3-indolyl phosphate (BCIP), LPS (from *Escherichia coli* O55:B5), 4′,6-diamidino-2-phenylindole (DAPI), and 6-carboxyfluorescein (6-CF) were purchased from Sigma-Aldrich (St. Louis, MO).

Anthra(1,9-*cd*)pyrazol-6(2*H*)-one (SP600125), (E)-3-(4-Methylphenylsulfonyl)-2-propenenitrile (BAY 11-7082), and cycloheximide were purchased from Enzo Life Sciences (Plymouth Meeting, PA). N-[(phenylmethoxy)carbonyl]-L-leucyl-N-[(1S)-1-formyl-3-methylbutyl]-L-leucinamide (MG132) and lactacystin were purchased from Cayman Chemical (Ann Arbor, MI). The primary antibodies were rabbit polyclonal antibodies against human Cx43, human NF-κB p50 (both from Santa Cruz Biotechnology, Santa Cruz, CA), human Nedd4, SAPK/JNK (Cell Signaling, Danvers, MA), or human glyceraldehyde-3-phosphate dehydrogenase (GAPDH; R&D Systems, Minneapolis, MN) or mouse monoclonal antibodies against human phospho-SAPK/JNK, bovine ubiquitin (P4D1, both from Cell Signaling), rat Cx43 (BD Transduction Laboratories, Lexington, KY), or pig glial fibrillary acidic protein (GFAP; Sigma-Aldrich). Texas red-conjugated horse anti-mouse IgG antibodies were purchased from Vector (Burlingame, CA), fluorescein isothiocyanate (FITC)-conjugated goat anti-rabbit IgG and horseradish peroxide (HRP)-conjugated goat anti-mouse IgG antibodies from Jackson ImmunoResearch Laboratories (Bar Harbor, ME), HRP-conjugated goat anti-rabbit IgG antibodies from Epitomics (Burlingame, CA), and alkaline phosphatase (AP)-conjugated goat anti-mouse IgG and anti-rabbit IgG antibodies from Promega (Madison, WI).

### Drug Treatments

LPS was dissolved in culture medium as a 1 mg/ml stock solution and cycloheximide was dissolved in water as a 20 mg/ml stock solution. Other stock solutions were made in DMSO: SP600125 and BAY 11-7082 at 20 mM, lactacystin at 5 mM, and MG132 at 10 mM. Primary rat astrocytes were grown to approximately 90% confluence, then were switched to fresh growth medium containing LPS (2 µg/ml) at 37°C for the indicated time. In experiments using the JNK inhibitor SP600125 (10 µM) or the NF-κB inhibitor BAY 11-7082 (5 µM), the inhibitor was added to the astrocytes for 30 min prior to the addition of LPS. It takes 30 min before these inhibitors start to work. To examine the effects of LPS on Cx43 protein degradation, the astrocytes were treated with LPS alone or together with cycloheximide (10 µg/ml), MG132 (5 µM), or lactacystin (5 µM); the appropriate concentration of sterile double-distilled water (0.05%) or DMSO (0.1%) was added to cultures as controls for cycloheximide or the other drugs, respectively. The cells were then processed for immunoprecipitation, immunoblotting, immunofluorescence microscopy, or 6-CF scrape loading analysis.

### Immunoprecipitation and Immunoblot Analysis

Whole cell lysates of primary astrocytes were prepared by sonication for 3×10 s on ice in 100 µl of lysis buffer [a modified RIPA buffer (50 mM Tris-HCl, pH 7.4, 1% NP-40, 150 mM NaCl, 1 mM EDTA) containing a protease inhibitor cocktail (1 mM PMSF and 1 µg/ml each of leupeptin, pepstatin, and aprotinin) and phosphatase inhibitors (1 mM NaF and 1 mM Na_3_VO_4_)]. For immunoprecipitation, all procedures were carried out at 4°C as described previously with minor modifications [21]. In brief, the cell lysate in a microtube was pre-cleared by adding 10 µl of Protein G-Sepharose 4 Fast Flow bead slurry (GE Healthcare, Piscataway, NJ) and incubating the suspension at 4°C for 30 min on a rocker. The Protein G-Sepharose beads were removed by centrifugation at 12,300×*g* for 10 min at 4°C. Equal amounts of protein sample (400 µg) and 1 µg of rabbit polyclonal antibodies against total Cx43 or normal rabbit serum (Santa Cruze Biotechnology) were mixed overnight at 4°C on a rocker, then the mixture was incubated for 2 h at 4°C with 100 µl of a slurry of Protein G-Sepharose. The Sepharose-bound immune complexes were then sedimented by centrifugation at 3,000×*g* for 5 min at 4°C and washed 4 times with 500 µl of lysis buffer, followed by centrifugation at 12,300×*g* for 1 min at 4°C. The pellets were resuspended in reducing Laemmli sample buffer (10% glycerol, 5% β-mercaptoethanol, 2% SDS, 0.003% bromophenol blue, 62.5 mM Tris-HCl, pH 6.8), boiled for 5 min at 100°C, centrifuged, and the supernatant collected.

Whole cell lysates and immunoprecipitates were boiled for 5 min, electrophoresed on 10% SDS-polyacrylamide gels, and transferred to Whatman Protran® nitrocellulose membranes (PerkinElmer Life and Analytical Sciences, Boston, MA). Strips of the membranes were blocked for 1 h at room temperature (RT) in blocking buffer [150 mM NaCl, 50 mM Tris-HCl, pH 7.4, 0.1% Tween-20 (TBST) containing 5% skim milk], then incubated overnight at 4°C with rabbit antibodies against NF-κB, Cx43, or Nedd4 or mouse antibodies against phospho-JNK or ubiquitin. After three washes with TBST, pH 7.4, the strips were incubated for 1 h at RT with AP-conjugated goat anti-rabbit IgG or goat anti-mouse IgG, followed by three washes with TBST, pH 8.2. Immunoreactive bands were developed using NBT and BCIP in 100 mM NaCl, 100 mM Tris-base, 5 mM MgCl_2_, pH 9.5. In membrane stripping experiments, the blots were stripped using 25 mM glycine-HCl, pH 2.0, 1% (w/v) SDS and reprobed with rabbit antiserum against GAPDH or total JNK or mouse antibodies against Cx43, followed by incubation with an HRP-conjugated secondary antibody. Bound antibodies were detected using Western Blotting Luminol Reagent (Santa Cruz Biotechnology) and Hyperfilm ECL (Amersham Pharmacia Biotech, Buckinghamshire, England, UK) before X-ray film exposure and development. The bands on nitrocellulose membranes and films were scanned and quantified using Gel-Pro Analyzer 3.1 software (Media Cybernetics, MD). All bands were normalized to GAPDH and expressed as percentage of control values.

### Immunofluorescence Microscopy

Primary astrocytes on collagen-coated glass coverslips were fixed in cold acetone for 10 min at −20°C and rinsed with phosphate-buffered saline (PBS) prior to incubation for 2 h at 37°C with a mixture of rabbit polyclonal antibodies against Cx43 and mouse monoclonal antibody against GFAP in PBS. After 3×5 min washes with PBS, the cells were incubated for 1 h at RT with a mixture of FITC-conjugated goat anti-rabbit IgG antibodies and Texas red-conjugated goat anti-mouse IgG antibodies in PBS. After 2×5 min washes with PBS and a brief rinse with 0.9% NaCl, the cells were incubated for 15 min at room temperature with a 1∶200 dilution of DAPI (2 mg/ml stock). After a brief rinse in 0.9% NaCl, they were mounted in Gel Mount™ aqueous mounting medium (Sigma-Aldrich) and sealed with nail polish. The stained astrocytes were examined using a Leica Microsystems microscope (Leica, Wetzlar, Germany) equipped for epifluorescence and images were acquired using a BD Confocal and Real-time Vision CARV II system (BD Bioscience).

### Scrape Loading and 6-CF Transfer Analysis

Scrape-loading/fluorescent dye transfer was performed to study functional coupling of the astrocyte cultures as described previously [14]. In brief, a scrape was made with a razor blade in a confluent layer of primary astrocytes in 35 mm culture dishes and the cells loaded with 6-CF at a concentration of 0.05% in PBS, then the cells were incubated for 2.5 min at RT. After 4 washes with PBS containing 0.9 µM CaCl_2_ and 1.05 µM MgCl_2_, the culture dishes were immediately transferred to a Leica DMI3000 B inverted microscope (Leica, Wetzlar, Germany) and images captured using a Canon 550D digital camera (Canon, Tokyo, Japan). The spread of 6-CF was measured by marking the farthest fluorescent cell borders on both sides of the scrape line and the fluorescent area in 10 randomly-selected non-overlapping fields along the scrape line (Fig. S2) quantified using Scion Image software (Frederick, MD).

### Statistical Analysis

All data are presented as the mean ± SD for at least three independent experiments. Statistical evaluation performed using Statistical Program for Social Sciences (SPSS) 16.0 (Inc, USA). The quantitative data of cycloheximide treatment were assessed using independent-sample *t*-test. All other experiments were assessed using one-way ANOVA with Dunnett’s post-hoc test. A *P* value <0.05 was considered statistically significant.

## Results

### LPS Upregulates Phospho-JNK and NF-κB Levels in Astrocytes

**Figure 1:**
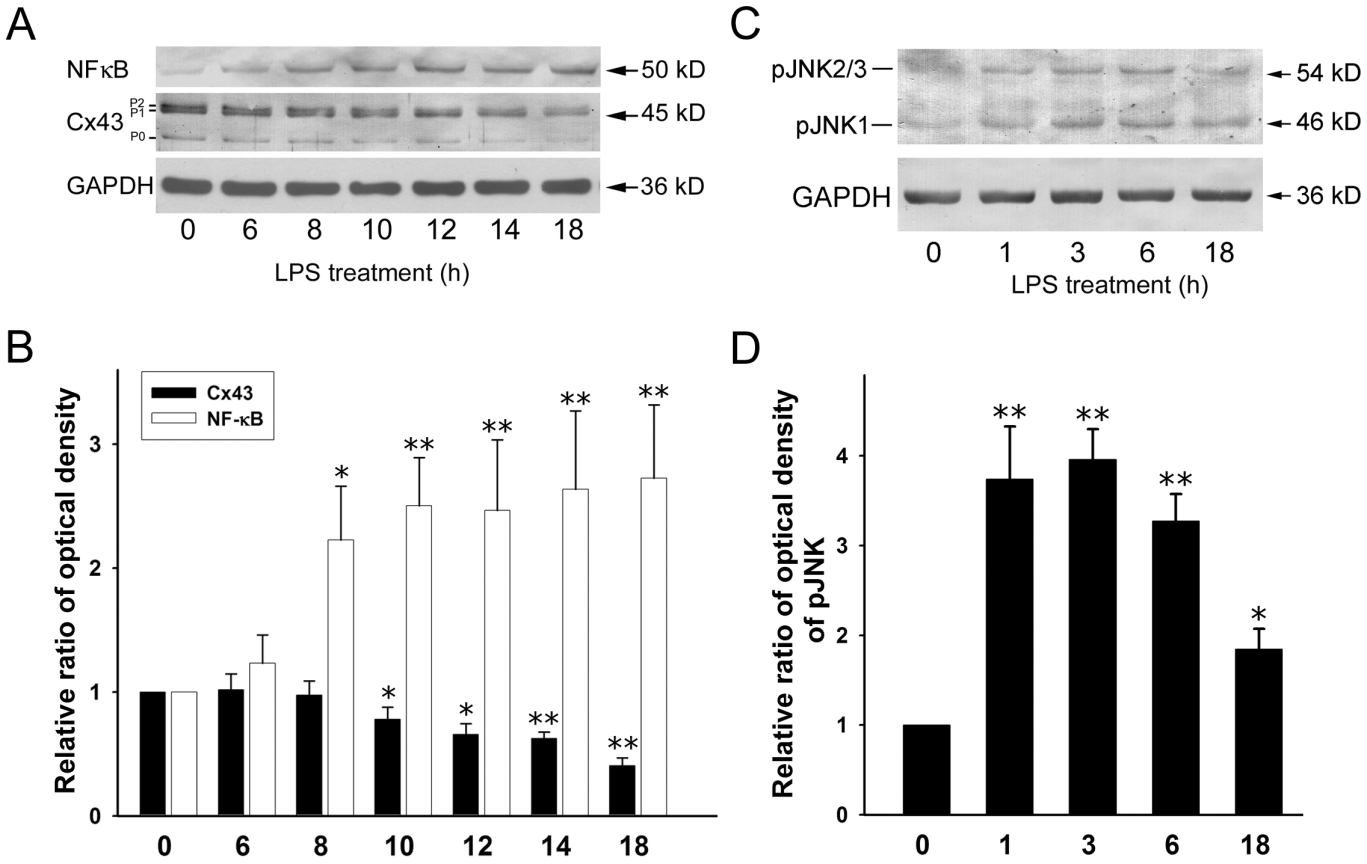
Time-dependent effects of LPS on levels of NF-κB, Cx43, and phospho-JNK in rat astrocytes. (**A**) Astrocytes were treated with 2 µg/ml of LPS for 0, 6, 8, 10, 12, 14, or 18 h, then whole cell lysates were collected, subjected to10% SDS-PAGE, and analyzed by immunoblotting with antibodies against NF-κB (**NF-κB**) or total Cx43 (**Cx43**). Glyceraldehyde 3-phosphate dehydrogenase (**GAPDH**) was used as the loading control. The three major bands of Cx43 are the non-phosphorylated form (P0) and the two phosphorylated forms (P1 and P2). (**B**) The blots from 4 independent experiments were subjected to densitometric analyses for total Cx43 (P0 plus P1 plus P2) and the results expressed as the density of the bands in the test sample relative to those in the time zero sample. *P<0.05, **P<0.01 compared to 0 h using Dunnett’s post-hoc test. (**C**) Whole cell lysates were prepared from astrocytes treated with 2 µg/ml LPS for 0, 1, 3, 6, or 18 h, subjected to 10% SDS-PAGE and immunoblotted with antibodies against the three isoforms (JNK1, JNK2, and JNK3) of phospho-JNK(**pJNK**) or GAPDH (**GAPDH**, loading control). (**D**) The blots from 3 independent experiments were subjected to densitometric analyses for phospho-pJNK (pJNK1 plus pJNK2/3) and the results expressed as the density of the bands in the test sample relative to those in the time zero sample. *P<0.05, **P<0.01 compared to 0 h using Dunnett’s post-hoc test.

Using MTT test, LPS concentrations ranging from 0.01 to 50 mg/ml did not affect astrocyte survival (Fig. S3). We first measured Cx43 protein levels in response to LPS in an immunoblot time-course study and found that Cx43 levels gradually decreased during treatment with LPS (2 µg/ml) for 10–18 h (Fig. 1A and B). We then examined whether LPS activated the JNK and NF-κB signaling pathways and as shown in Fig. 1A (top panel), only low levels of NF-κB were found in control astrocytes, but a significant increase in NF-κB levels was detected at 6–18 h of LPS treatment (Fig. 1A and B). In contrast, a transient increase in pJNK-1, -2, and -3 levels was detected at 1–6 h of LPS treatment, and still increased at 18 h but significantly less than 1–6 h (Fig. 1C and D).

### The JNK Inhibitor SP600125 Inhibits LPS-induced Cx43 Downregulation in Astrocytes

**Figure 2:**
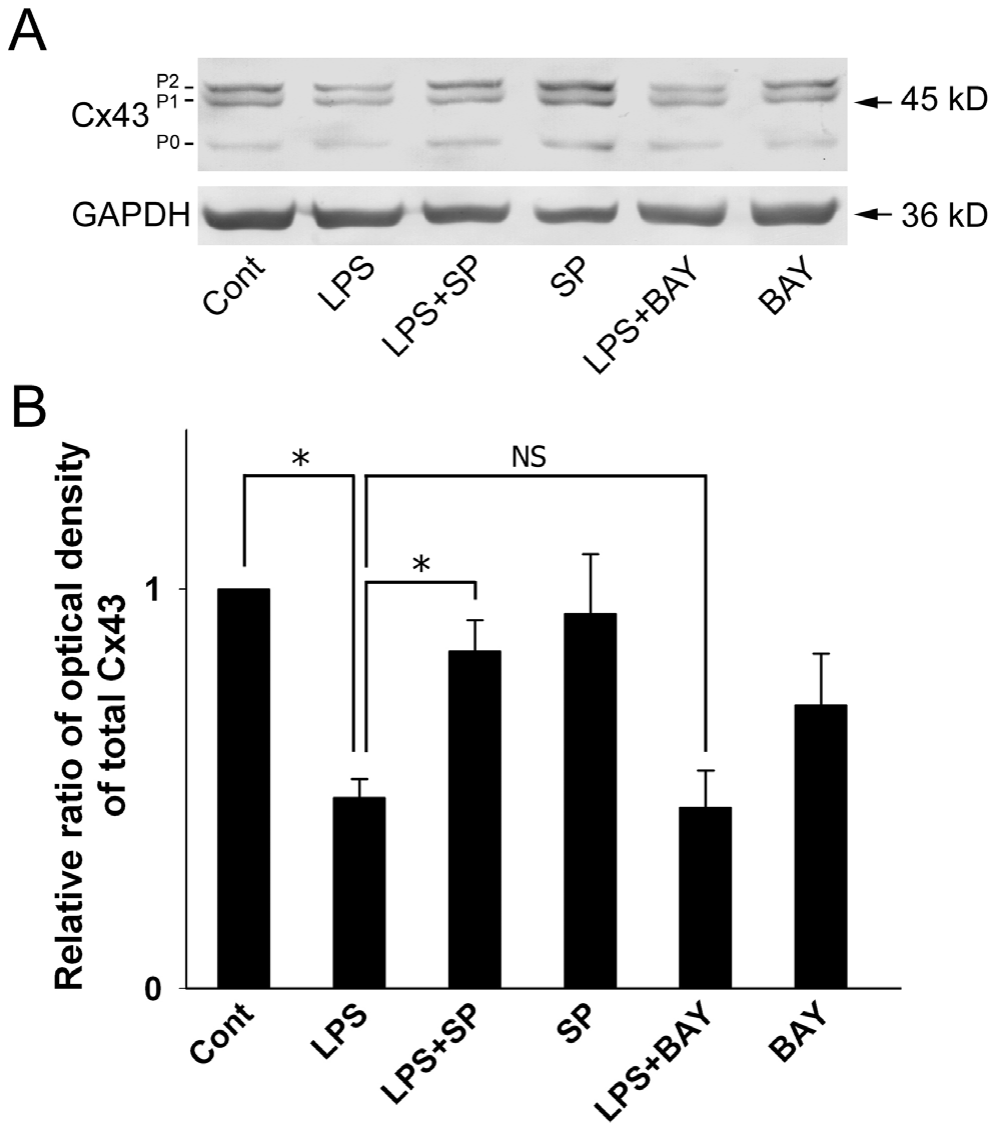
The JNK inhibitor SP600125 inhibits LPS-induced downregulation of Cx43 in astrocytes. (**A**) Astrocytes were treated for 18 h with 0.05% DMSO (**Cont**), 2 µg/ml of LPS (**LPS**), 10 µM SP600125 (**SP**), or 5 µM BAY11-7082 (**BAY**) or were treated with SP600125 (**LPS**+**SP**) or BAY11-7082 (**LPS**+**BAY**) for 30 min prior to, and during, incubation with LPS for 18 h. Cell lysates were prepared and total proteins analyzed by 10% SDS-PAGE and immunoblotting using antibodies against total Cx43 or GAPDH (**GAPDH**, loading control). (**B**) The blots from 3 independent experiments were subjected to densitometric analyses for total Cx43 (P0 plus P1 plus P2) and the results expressed as the density of the bands in the test sample relative to those in the control. *P<0.01, NS (not significant) compared to the LPS-treated group using Dunnett’s post-hoc test.

LPS treatment induced a significant decrease in Cx43 levels compared to controls (Fig. 2, LPS) and this effect was inhibited by pretreatment with SP600125 (Fig. 2, LPS+SP), but not BAY11-7082 (Fig. 2, LPS+BAY).

### SP600125 Prevents LPS-induced Gap Junction Disassembly and GJIC Inhibition in Astrocytes

**Figure 3:**
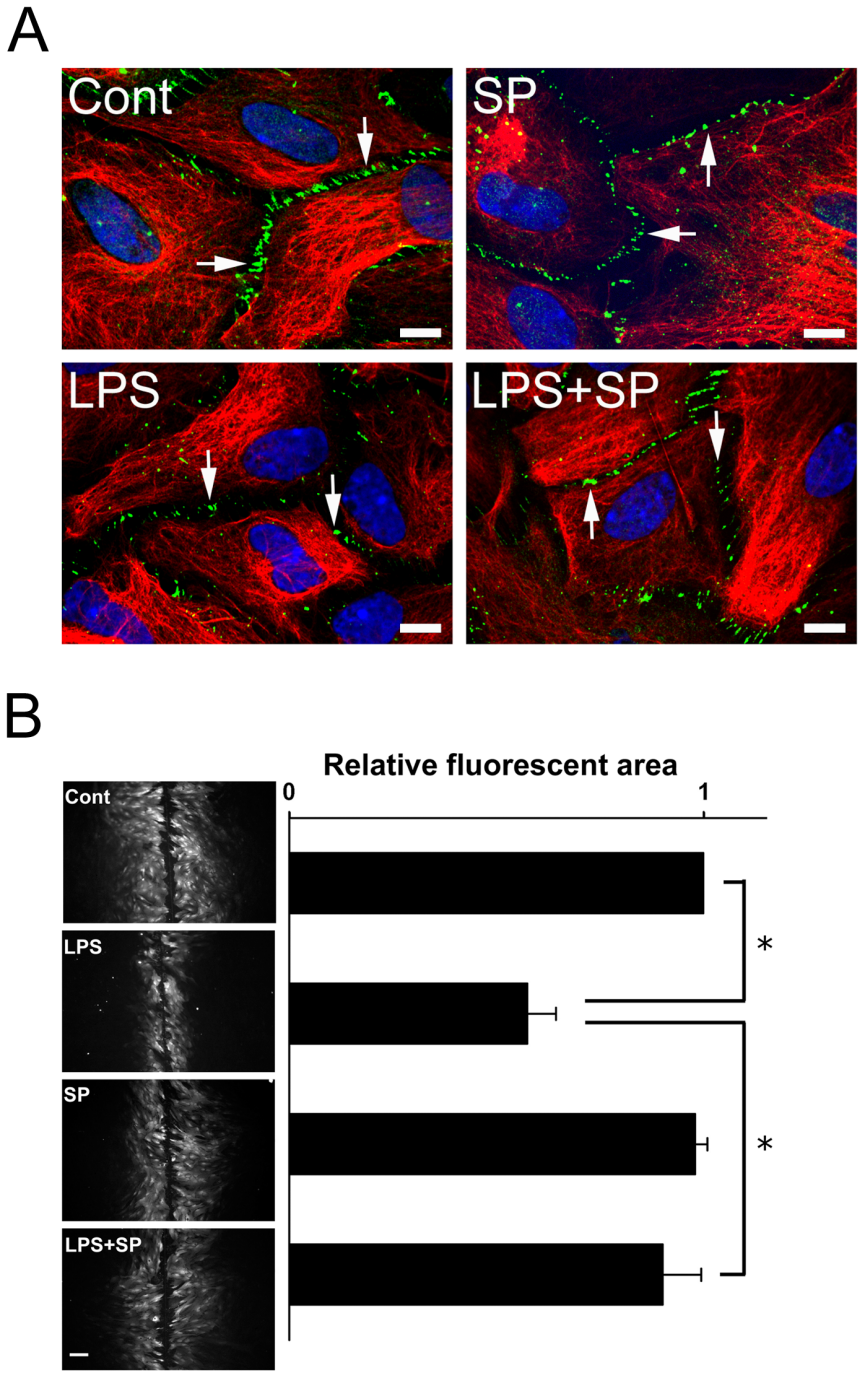
Effects of SP600125 on Cx43 gap junction plaque staining and 6-CF fluorescent dye transfer in LPS-treated rat astrocytes. (**A**) Astrocytes were treated for 18 h with 0.05% DMSO (**Cont**), 2 µg/ml of LPS (**LPS**), 10 µM SP600125 (**SP**), or 5 µM BAY11-7082 (**BAY**) or were treated with SP600125 (**LPS**+**SP**) or BAY11-7082 (**LPS**+**BAY**) for 30 min prior to, and during, incubation with LPS for 18 h. The confocal microscopy merged images show control or treated astrocytes double-immunostained with antibodies against Cx43 (**Cx43**, green) and glial fibrillary acidic protein (**GFAP**, red). Nuclei were stained with **DAPI** (blue). The arrows indicate Cx43 gap junction plaques at cell-cell contact points. Bar = 10 µm. (**B**) Astrocytes were treated for 18 h with 0.05% DMSO (**Cont**) or 2 µg/ml of LPS (**LPS**) or were preteated for 30 min with 10 µM SP600125 followed by treatment for 18 h in absence of LPS (**SP**) or were pretreated for 30 min with SP600125 followed by LPS treatment for 18 h in the continued presence of the inhibitor (**LPS+SP**) subjected to 6-CF fluorescent dye scrape loading as described in the Materials and Methods (**left panels**, bar = 100 µm). The right panel shows the data for the dye-spreading area from 3 independent experiments expressed as the fluorescent area relative to that in the control. *P<0.01 compared to the LPS-treated group using Dunnett’s post-hoc test.

Astrocytes were double-immunostained for Cx43 and glial fibrillary acidic protein (GFAP) and visualized by confocal microscopy. The merged images showed extensive punctate Cx43 staining of gap junction plaques at contiguous plasma membranes in control astrocytes (Fig. 3A, Cont), whereas LPS treatment induced dispersed Cx43 staining, indicating gap junction disassembly (Fig. 3A, LPS). Pretreatment with the JNK inhibitor SP600125 inhibited the effect of LPS on Cx43 staining at cell-cell contacts (Fig. 3A, LPS+SP). SP600125 treatment alone only had little effect on Cx43 staining (Fig. 3A, SP). Scrape-loading analysis of 6-CF was then used to assess GJIC by fluorescent dye spreading in astrocytes. Considerable dye transfer was observed in control astrocytes (Fig. 3B, Cont), whereas LPS treatment caused a significant decrease in dye spreading (Fig. 3B, LPS) and this effect was inhibited by pretreatment with SP600125 (Fig. 3B, LPS+SP). SP600125 treatment alone had no effect on fluorescent dye spreading (Fig. 3B, SP).

### LPS Induces Cx43 Ubiquitination and Accelerates Cx43 Degradation in Astrocytes

**Figure 4:**
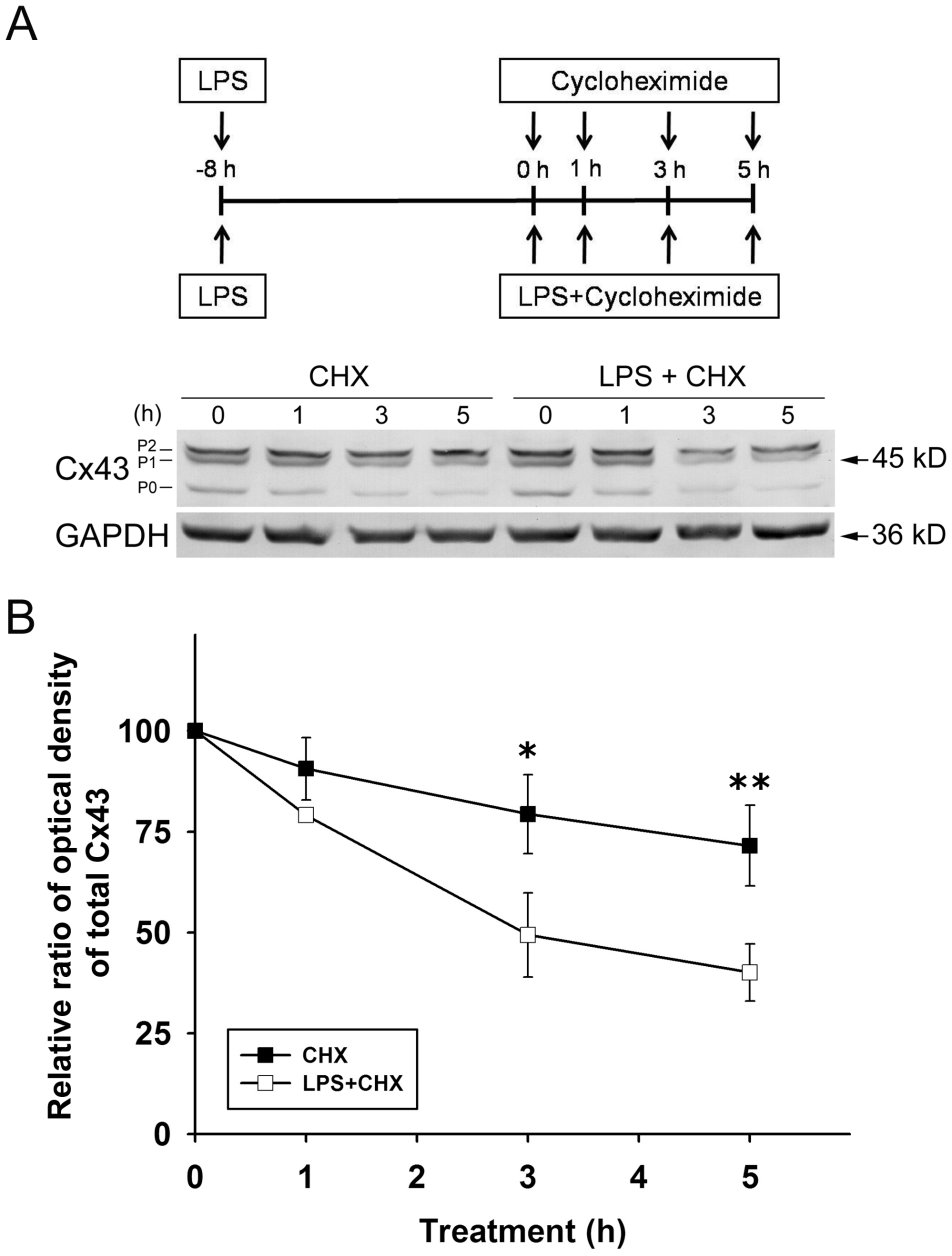
LPS accelerates Cx43 degradation in cycloheximide-treated astrocytes. (**A**) Astrocytes were pretreated with 2 µg/ml LPS (**LPS**) for 8 h followed by treatment for 1, 3, or 5 h with 10 µg/ml of cycloheximide in the absence (**CHX**) or in the continued presence of LPS (**LPS+CHX**), then the cells were lysed and the whole cell lysates subjected to10% SDS-PAGE, and analyzed by immunoblotting with antibodies against total Cx43 (**Cx43**, P0, P1, and P2). The blots were then stripped and reprobed with antibodies against GAPDH (**GAPDH**, loading control). (**B**) The blots from 3 independent experiments were subjected to densitometric analyses for total Cx43 (P0 plus P1 plus P2) and the results expressed as the density of the bands in the test sample relative to those in the time zero sample of **CHX** or **LPS+CHX**. *P<0.05, **P<0.01 compared to CHX using independent-sample *t*-test.

**Figure 5:**
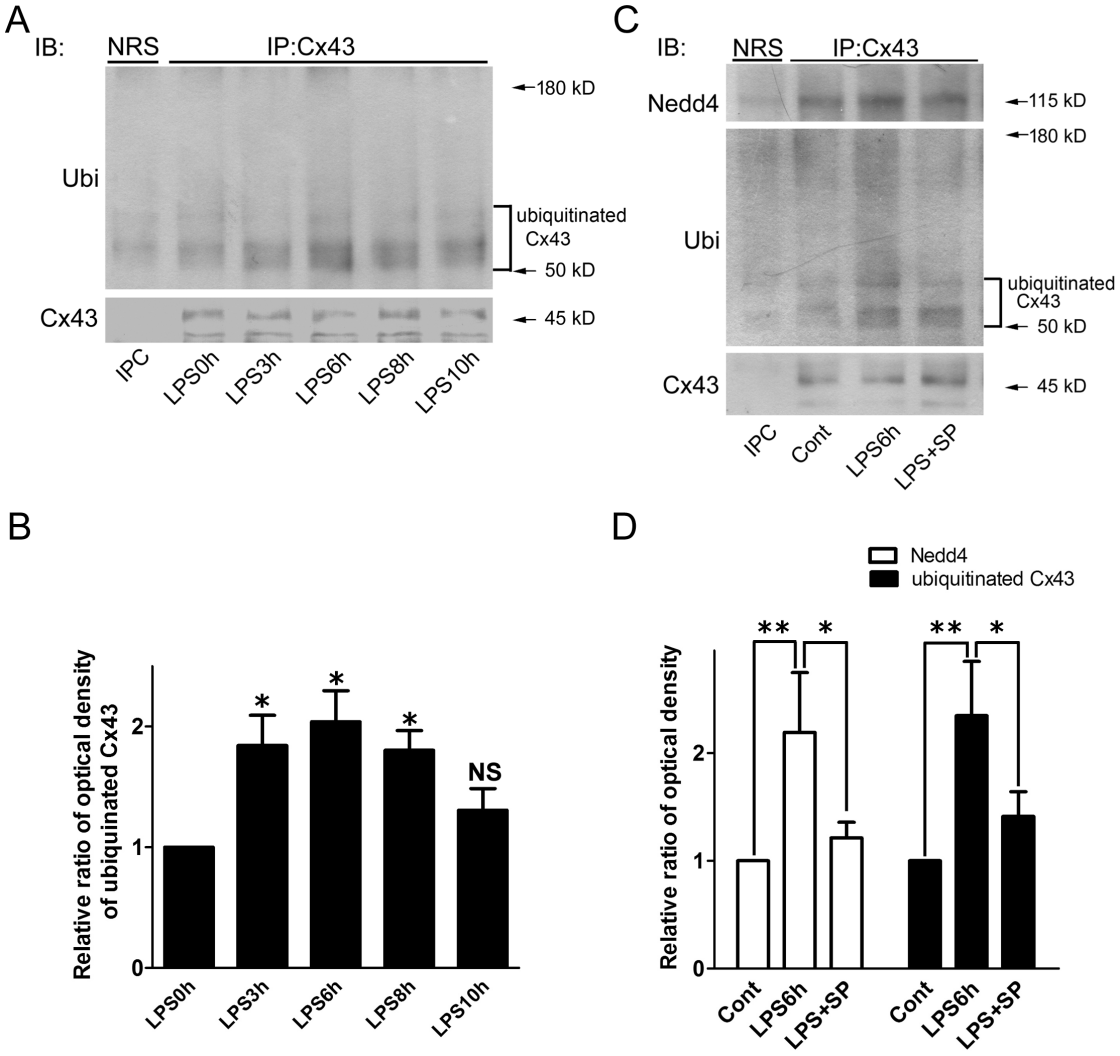
SP600125 prevents Cx43 ubiquitination in LPS-treated rat astrocytes. (**A**) Astrocytes were treated with 2 µg/ml of LPS for 0, 3, 6, 8, or 10 h. The cell lysates immunopreciptated using normal rabbit serum (**NRS,** immunoprecipitation control, **IPC**) or rabbit antibody against total Cx43 (**IP: Cx43**). The immunoprecipitates were then subjected to 10% SDS-PAGE and immunoblotting using antibodies against ubiquitin (**IB: Ubi**). The immunoprecipitates were then subjected to 10% SDS-PAGE and immunoblotting using antibodies against ubiquitin (**IB: Ubi**) or total Cx43 (**IB: Cx43**). (**B**) The blots from 3 independent experiments were subjected to densitometric analyses for ubiquitinated Cx43 and the results expressed as the density of the bands in the test sample relative to those in the time zero sample. *P<0.05, NS (not significant) compared to 0 h using Dunnett’s post-hoc test. (**C**) The cell lysates from astrocytes treated with 0.05% DMSO (**Cont**), 2 µg/ml of LPS (**LPS**) for 6 h, or were pretreated for 30 min with 10 µM SP600125 followed by LPS (**LPS+SP**) treatment for 6 h in the presence of the inhibitor were immunopreciptated using normal rabbit serum (**NRS,** immunoprecipitation control, **IPC**) or rabbit antibody against total Cx43 (**IP: Cx43**). The immunoprecipitates were then subjected to 10% SDS-PAGE and immunoblotting using antibodies against ubiquitin (**IB: Ubi**), Nedd4 (**IB: Nedd4**) or total Cx43 (**IB: Cx43**). (**D**) The blots from 3 independent experiments were subjected to densitometric analyses for Nedd4 or ubiquitinated Cx43 and the results expressed as the density of the bands in the test sample relative to those in the control. *P<0.05, **P<0.01 compared to the LPS-treated group using Dunnett’s post-hoc test.

To investigate whether LPS plays a role in the degradation of Cx43, astrocytes were pretreated with LPS, then they were incubated with cycloheximide to block de novo protein synthesis in the absence or continued presence of LPS (Fig. 4). Cycloheximide exposure alone for 1–5 h caused a time-dependent decrease in Cx43 levels, indicating gradual Cx43 degradation in cycloheximide-treated astrocytes (Fig. 4, CXH) and this effect was accelerated by LPS co-treatment (Fig. 4, LPS+CHX). Since ubiquitination has been reported to be an important signal regulating Cx43 internalization from the plasma membrane and its subsequent degradation by the proteasomal proteolytic pathway [22], we next tested the effect of LPS on ubiquitination of Cx43 in astrocytes using immunoprecipitation with a rabbit polyclonal antibody against total Cx43, followed by immunoblotting of the precipitated proteins with a monoclonal anti-ubiquitin antibody. As shown in Figure 5, in spite of a slight association between Cx43 and ubiquitin in control astrocytes (Fig. 5A, 2^nd^ lane; Fig. 5B), LPS treatment for 3–8 h promoted the association between Cx43 and ubiquitin and induced an increase in ubiquitinated Cx43 levels (Fig. 5A, 3^rd^, 4^th^ and 5^th^ lanes; Fig. 5B). LPS treatment also promoted the binding of E3 ubiquitin ligase Nedd4 to Cx43 (Fig. 5C, upper panel, 3^rd^ lane; Fig. 5D) and this effect was inhibited by pretreatment with the JNK inhibitor SP600125 (Fig. 5C, upper panel, 4^th^ lane; Fig. 5D). The LPS-induced increase in ubiquitinated Cx43 levels was also inhibited by pretreatment with SP600125 (Fig. 5C, middle panel, 4^th^ lane; Fig. 5D). Total levels of ubiquitin were unchanged in LPS-treated astrocytes in the absence or presence of SP600125 (data not shown).

### The Proteasome Inhibitors MG132 and Lactacystin Prevent LPS-induced Cx43 Degradation

**Figure 6:**
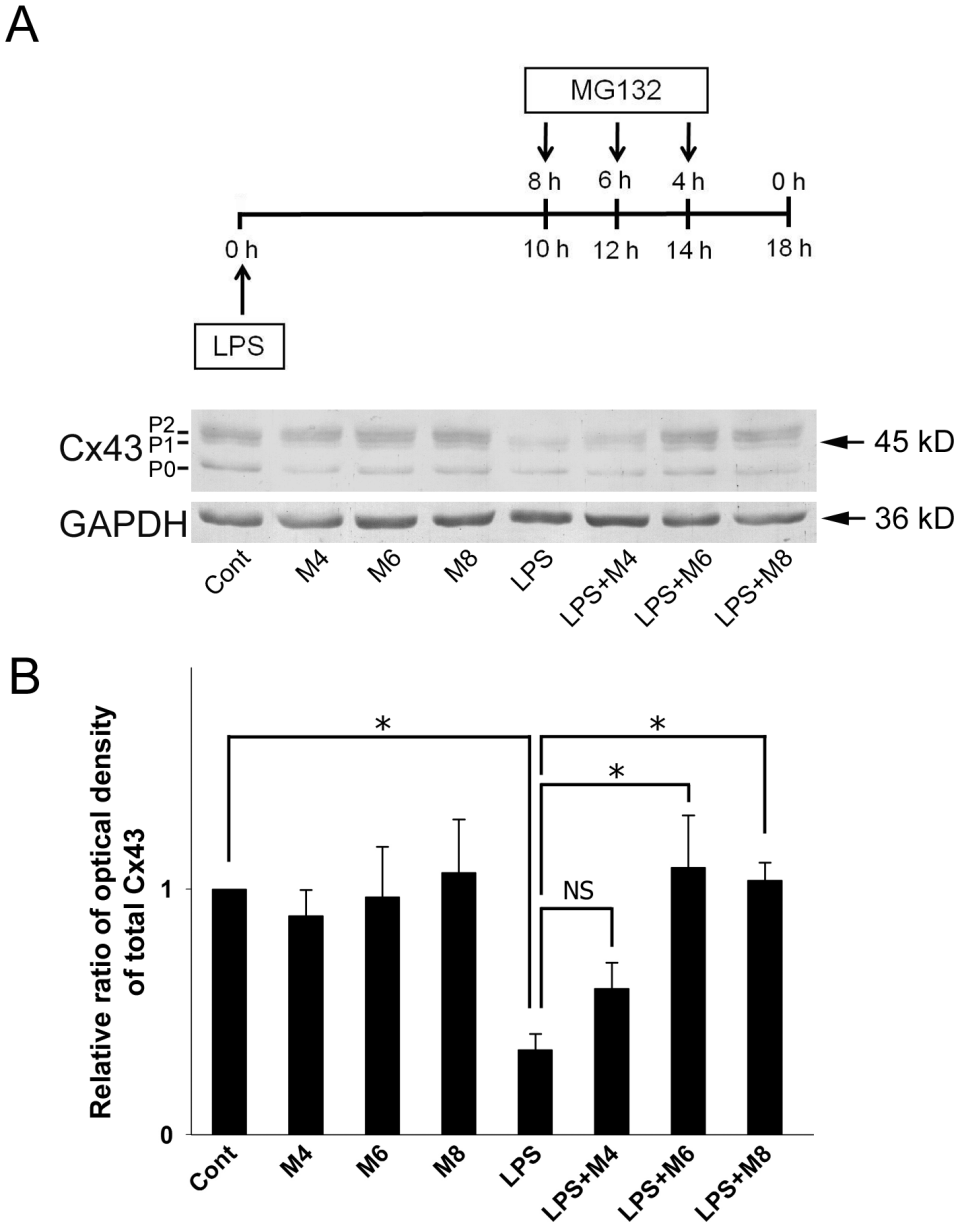
Effect of the proteasome inhibitor MG132 on Cx43 levels in LPS-treated astrocytes. (**A**) Astrocytes were treated with 0.05% DMSO for 8 h (**Cont**), with 5 µM MG132 for 4, 6, or 8 h (**M4, M6, M8**), with 2 µg/ml of LPS for 18 h (**LPS**), or with LPS for 18 h with addition of MG132 for the last 4, 6, or 8 h (**LPS+M4, LPS+M6, LPS+M8**), then cell lysates were prepared, subjected to 10% SDS-PAGE, and analyzed by immunoblotting with antibodies against total Cx43 (**Cx43**) or GAPDH (**GAPDH**, loading control). (**B**) Densitometric analyses of total Cx43 (P0 plus P1 plus P2) from 3 independent experiments expressed as the density of the bands in the test sample relative to those in the control. *P<0.01, NS (not significant) compared to the LPS-treated group using Dunnett’s post-hoc test.

**Figure 7:**
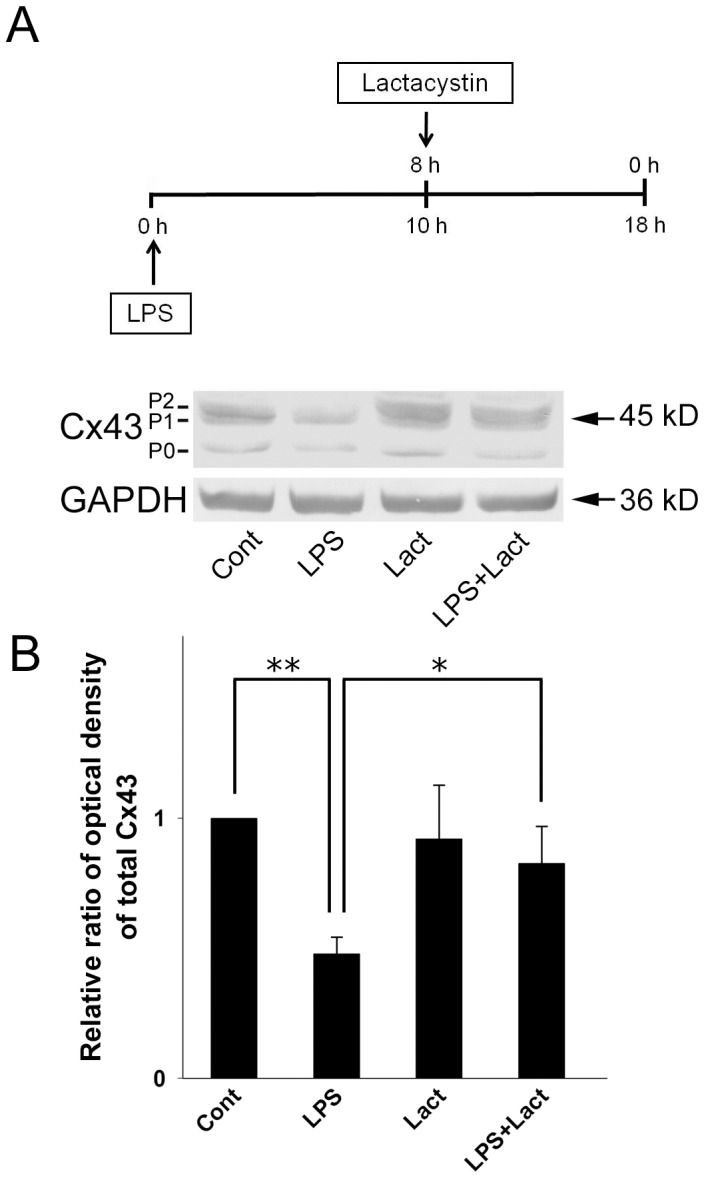
Effect of the proteasome inhibitor lactacystin on Cx43 levels in LPS-treated astrocytes. (**A**) Astrocytes were treated for 8 h with 0.1% DMSO (**Cont**), for 18 h with 2 µg/ml of LPS (**LPS**), for 8 h with 5 µM lactacystin (**Lact**), or with 2 µg/ml LPS for 18 h with addition of lactacystin for the last 8 h (**LPS**+**Lact**), then cell lysates were prepared, subjected to 10% SDS-PAGE, and analyzed by immunoblotting with antibodies against total Cx43 (**Cx43**, P0, P1, and P2) or GAPDH (**GAPDH**, loading control). (**B**) Densitometric analyses of total Cx43 (P0 plus P1 plus P2) from 3 independent experiments expressed as the density of the bands in the test sample relative to those in the control. **P<0.01, *P<0.05 compared to the LPS-treated groups using Dunnett’s post-hoc test.

We used specific proteasome pathway inhibitors to clarify whether the LPS-induced degradation of Cx43 was mediated by the proteasome. In a time-course study, stimulation with LPS for 18 h in the absence of any inhibitor caused a dramatic decrease in Cx43 levels (Fig. 6, LPS) and this effect was significantly inhibited by addition of the reversible proteasome inhibitor MG132 for the last 4–8 h of LPS incubation (Fig. 6, LPS+M4, LPS+M6, LPS+M8). Although treatment with MG132 alone caused a slight increase in phospho-Cx43 (P1 and P2), total Cx43 levels were unchanged compared to the controls (Fig. 6, M4, M6, M8). We also used an irreversible inhibitor, lactacystin, to block the proteasome proteolytic pathway and, as shown in Figure 7, the Cx43 degradation induced by 18 h of LPS treatment (Fig. 7, LPS) was blocked by addition of lactacystin for the last 8 h (Fig. 7, LPS+Lact).

## Discussion

In the present study, LPS treatment was shown to induce JNK activation and NF-κB upregulation in primary rat astrocytes. Pretreatment with the JNK inhibitor SP600125, but not the NF-κB inhibitor BAY11-7082, blocked the inhibitory effect of LPS on Cx43 protein expression and GJIC, demonstrating a role of the JNK signaling pathway in the LPS-induced effects on the gap junction. A previous study demonstrated that activation of the NF-κB and PI3 kinase pathways, but not JNK pathway, by TLR3 leads to a reduction in Cx43 expression in astrocytes [12]. When NF-κB activation is blocked using the specific pharmacological I-κB kinase (IKKβ) inhibitor IKKβ inhV or NF-κB essential modulator (NEMO)-deficient mutants, this fails to prevent the TNF-α-induced inhibition of Cx43 expression and GJIC [23]. Conversely, the JNK inhibitor SP600125 or the use of JNK-deficient mutants abolishes the TNF-α-induced inhibitory effect on Cx43 expression and GJIC in human HaCat keratinocytes [23]. Zang et al. (2013) reported that treatment of spinal primary astrocytes for 30 min with a mixture of TNF-α and IFN-γ induced the phosphorylation of JNK and its downstream effector c-Jun, whereas reduction of Cx43 mRNA levels was observed after prolonged treatment of both cytokines for 3 h, leading to the reduction of the protein levels of Cx43 and GJIC [24]. These effects were prevented by JNK inhibitor SP600125, suggesting that both cytokine-inhibited Cx43 expression and GJIC may be mediated by JNK-c-Jun pathway [24]. Here, our present study provided another regulatory role of JNK pathway in LPS-induced Cx43 degradation by ubiquitin-proteasome proteolysis.

We have previously shown that short-term LPS treatment (1–6 h) does not alter Cx43 protein levels, while long-term treatment (18–72 h) dramatically reduces Cx43 levels [14]. In the present study, LPS treatment for 10–18 h caused a time-dependent decrease in Cx43 protein levels in astrocytes. Gap junction channels are constituted of highly dynamic plasma membrane connexin proteins, which have rapid turnover rates ranging from 1.5 to 5 h [25], [26]. The use of the protein synthesis inhibitor cycloheximide to block storage of Cx43 in the Golgi apparatus showed that cycloheximide treatment for 2 h causes a decrease in Cx43-P0 levels [22]. In accordance with this result, we showed that levels of Cx43-P0 and total Cx43 gradually decreased with cycloheximide treatment. Moreover, coexposure of astrocytes to LPS and cycloheximide induced a dramatic decrease in Cx43 levels, indicating that LPS is able to accelerate Cx43 degradation. In a previous study, Esen et al. (2007) showed that both *Staphylococcus aureus* and PGN stimuli cause a decrease in astrocytic Cx43 and Cx30 expression concomitant with the inhibition of GJIC, whereas Cx26 levels are increased in response to these bacterial stimuli. Furthermore, inhibition of de novo protein synthesis by cycloheximide had no effect on the ability of *Staphylococcus aureus* to inhibit astrocyte GJIC, implying that bacterial stimuli regulates GJIC via direct effects [13]. Studies using specific pharmacological inhibitors of the lysosomal and proteasomal pathways have shown that both pathways participate in the degradation of Cx43 [17]–[19], [22], [27]. Our present study showed that downregulation of Cx43 expression in LPS-treated astrocytes was blocked by the proteasome inhibitors MG132 and lactacystin, showing that LPS induces Cx43 degradation by the proteasome proteolytic pathway.

Several studies have demonstrated that the proteolytic degradation of Cx43 required its post-translational modification by ubiquitin. In serum-deprived corneal fibroblasts, TNF-α treatment for 5 min induces increased ubiquitination of Cx43, while prolonged treatment for 12–24 h results in a dramatic decrease in Cx43 protein levels [19]. Our present study showed that primary astrocytes expressed ubiquitinated proteins, including Cx43. Although ubiquitin levels remained unchanged after LPS treatment for 6 h, increased association of Cx43 and ubiquitin was detected by co-immunoprecipitation using anti-ubiquitin antibody P4D1, which recognizes poly- and monoubiquitinated proteins [27]. Our immunoblot analyses revealed that multiple bands of ubiquitinated Cx43 migrate between 50 and 70 kD (Fig. 5), implying that Cx43 may be subjected to monoubiquitination after LPS treatment. Thus, LPS-induced Cx43 degradation in astrocytes might be mediated by multi-monoubiquitination and the ubiquitin-proteasome pathway, which would be a highly non-canonical pathway [28].

It has been suggested that ubiquitin-interacting proteins have regulatory effects on the internalization and post-endocytic trafficking of ubiquitinated Cx43. For example, the binding of the E3 ubiquitin ligase Nedd4 to Cx43 results in Cx43 monoubiquitination and its interaction with the endocytic adaptor Eps15, leading to Cx43 displacement from the plasma membrane [29]. A recent study on rat liver epithelial IAR20 cells demonstrated that conjugation of Cx43 gap junction plaques with ubiquitin by recruiting the ubiquitin-binding proteins Hrs and Tsg101 regulates Cx43 endocytic trafficking and sorting [15], while, in human corneal fibroblasts subjected to inflammatory stimuli, TNF-α induces a rapid association of Nedd4 and ubiquitin with Cx43, which is subsequently degraded by the proteasome [19]. These findings are consistent with our present study showing that LPS promoted Cx43 ubiquitination via the association of Cx43 with ubiquitin and Nedd4, which might subsequently accelerate the turnover of Cx43.

Several lines of evidence indicate that JNK-mediated phosphorylation plays a key role in regulating the ubiquitination and stability of target proteins. As shown by our immunoprecipitation analyses, the LPS-induced increased interaction between ubiquitin and Cx43 was partially blocked by the JNK inhibitor SP600125, indicating that LPS promotes Cx43 ubiquitination via activation of the JNK pathway and recent studies have shown that phosphorylation of the transcription factor MafB and nuclear retinoic acid receptor-α by JNK results in their degradation by ubiquitination and proteolysis [30], [31]. In contrast, other studies have suggested that JNK-mediated phosphorylation of transcription factor SP1 and tumor suppressor P53 leads to inhibition of ubiquitination and protects these proteins from ubiquitin-dependent degradation [32], [33]. Previous studies focused on the activation of JNK have indicated a pivotal role of JNK in negatively regulating Cx43 protein levels [23], [34], [35]. Although other studies have suggested that EGF-induced disruption of GJIC is mediated by phosphorylation of serine residues on the C-terminal of Cx43 by MAPK [36]–[38], there is no evidence that JNK can directly phosphorylate Cx43. How JNK-mediated ubiquitination is involved in the LPS-induced degradation of Cx43 requires further investigation.

The involvement of JNK-mediated signaling pathway in CNS-associated diseases has been reported in several previous studies. Astrocyte-derived S100 protein induced JNK phosphorylation, contributing to neurofibrillary tangles (NFTs) formation in Alzheimer's disease (AD) [39]. JNK activation by IL-1β or *Mycobacterium tuberculosis* increases matrix metalloproteinase-9 expression in rat brain astrocytes, suggesting this effect may be involved in the neuroinflammatory processes [40], [41]. In addition, a previous study have demonstrated that JNK and its downstream effector c-Jun play an important role in neurodegenerative diseases by upregulation of cyclooxygenase-2 expression and endoplasmic reticulum stress in astrocytes [42]. Dysfunction of Cx43 gap junction was also considered to involve in the pathology of neurodegenerative or neuroinflammatory diseases such as Parkinson’s disease, multiple sclerosis, experimental allergic encephalomyelitis and Pelizaeus-Merzbacher-like disease [43]–[46]. Here, we showed that activation of JNK by LPS contributes to a reduction of Cx43 expression and GJIC in astrocytes via ubiquitin-proteasome pathway, suggesting that inhibition of JNK signaling cascades may provide therapeutic benefit in the development of CNS disorders.

**Figure 8:**
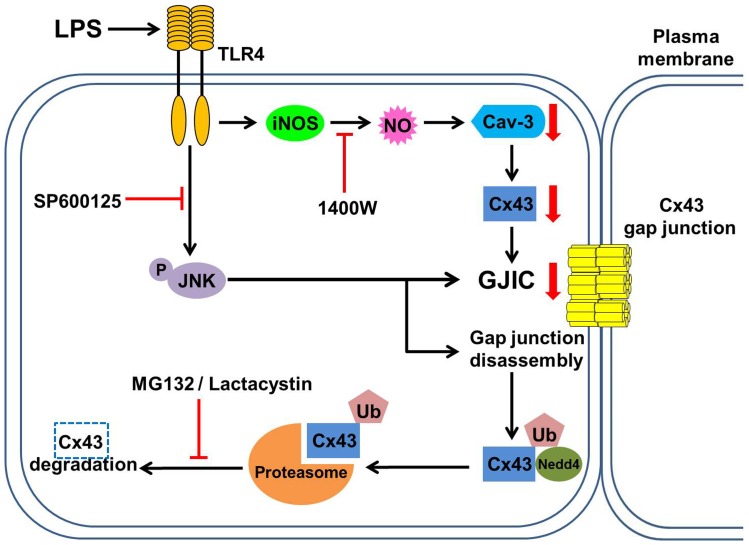
Schematic illustration of the signaling pathway involved in the inhibitory effect of LPS on Cx43 gap junctions in rat astrocytes. LPS treatment down-regulates caveolin-3 levels via TLR4-mediated iNOS activation, which in turn reduces the expression of Cx43. LPS also induces JNK phosphorylation, resulting in GJIC inhibition, gap junction disassembly, and Cx43 degradation by the ubiquitin-proteasome proteolytic pathway.

In conclusion, we have previously reported that LPS induces TLR4 activation, iNOS upregulation and caveolin-3 downregulation, leading to the inhibition of Cx43 expression and GJIC [14]. In the present study, we have demonstrated that activation of SAPK/JNK and the ubiquitin-proteasome proteolytic pathway by LPS results in decreased Cx43 expression and thus hinds astrocytic GJIC (Fig. 8). LPS treatment also causes an increased interaction between Cx43, Nedd4, and ubiquitin. Further studies on the role of JNK in Cx43 ubiquitination and how proteasomes regulate Cx43 degradation will help in elucidating how Cx43 gap junction turnover is regulated in astrocytes under inflammatory conditions.

## Supporting Information

Figure S1 The purity of primary astrocyte cultures. Confluent primary astrocytes were immunostained for glial fibrillary acidic protein (**GFAP**, red) and counterstained with **DAPI** (blue) for nuclei. The arrows indicate the GFAP-negative cells. Bar = 20 µm.
(TIF)

Figure S2 Schematic illustration of the fluorescent area measurement. After confluent astrocytes were incubated with 6-CF fluorescent dye, the photomicrographs of 10 randomly-selected fields from scrape lines were taken. The fluorescent area between the farthest cell borders on both sides of the scrape line (white dotted line) was measured.
(TIF)

Figure S3 Dose-dependent effect of LPS on astrocyte viability. Control astrocytes (**Cont**) or astrocytes treated for 24 h with 0.01, 0.1, 1, 10 or 50 µg/ml LPS were subjected to the MTT assay. Cells were also exposed to 3% or 5% DMSO or 1 mM H_2_O_2_ as positive control. The upper panel shows the MTT colorimetric assay. The lower panel shows the quantitative data for the MTT test from 3 independent experiments expressed as A570 nm relative to the control. *p<0.01 compared to the control group.
(TIF)
