# Supplementary figures and images for: Lipopolysaccharide Induces Degradation of Connexin43 in Rat Astrocytes via the Ubiquitin-Proteasome Proteolytic Pathway
Source: PLoS One. 2013 Nov 13;8(11):e79350. doi: 10.1371/journal.pone.0079350 (PMC3827358; doi:10.1371/journal.pone.0079350)

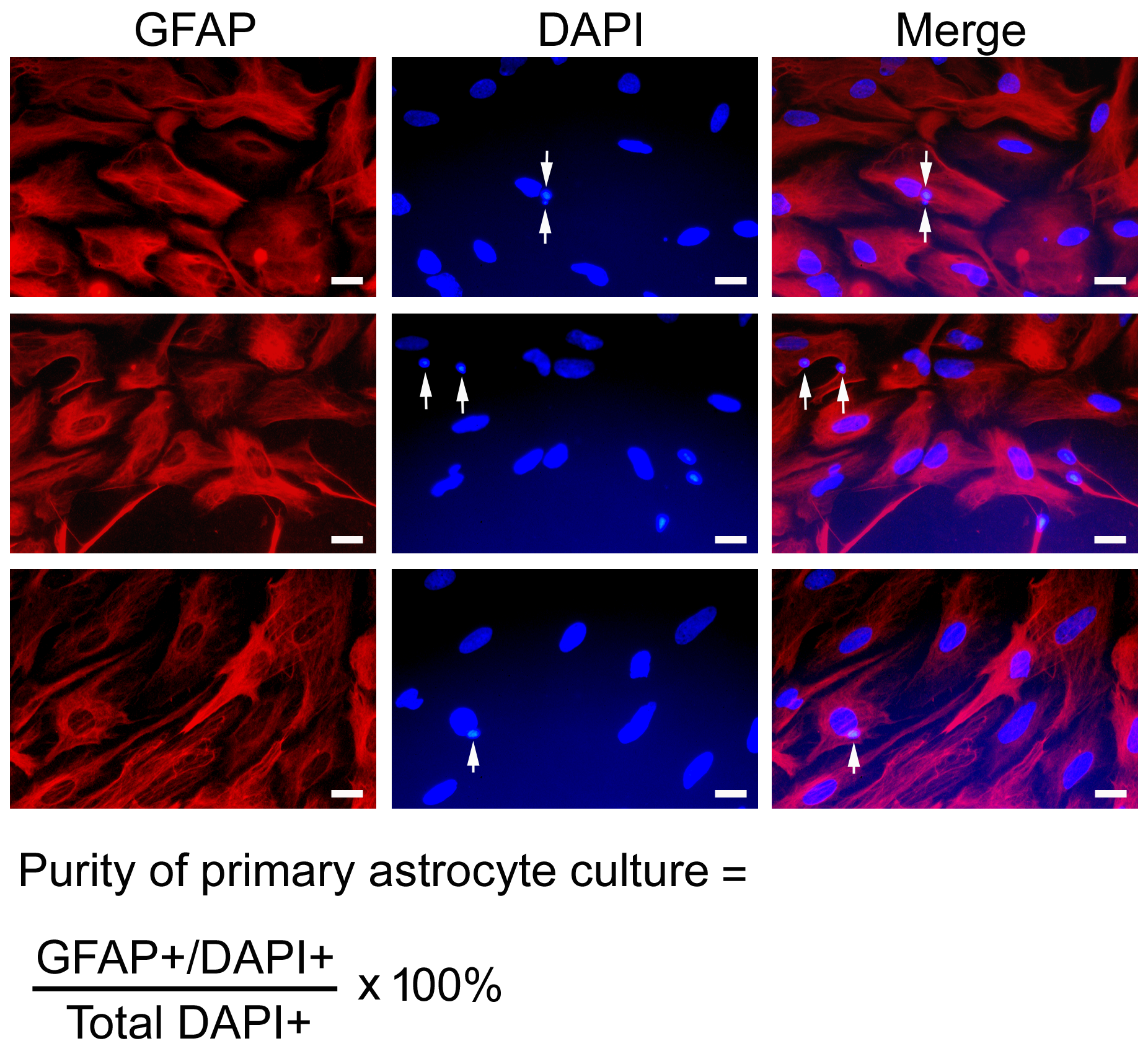

Supplement: Figure S1 — The purity of primary astrocyte cultures. Confluent primary astrocytes were immunostained for glial fibrillary acidic protein (GFAP, red) and counterstained with DAPI (blue) for nuclei. The arrows indicate the GFAP-negative cells. Bar = 20 µm. (TIF) [file pone.0079350.s001.tif]

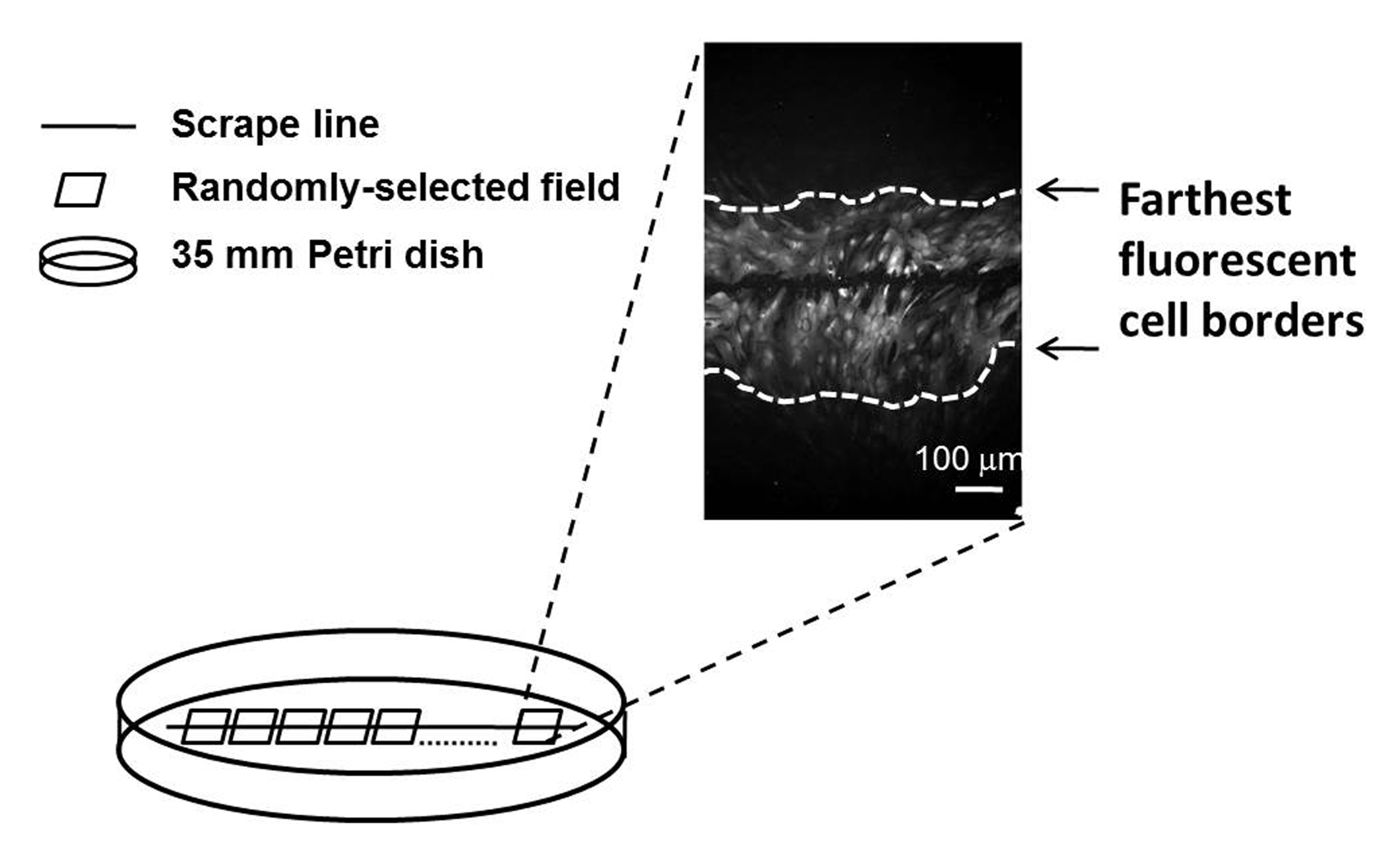

Supplement: Figure S2 — Schematic illustration of the fluorescent area measurement. After confluent astrocytes were incubated with 6-CF fluorescent dye, the photomicrographs of 10 randomly-selected fields from scrape lines were taken. The fluorescent area between the farthest cell borders on both sides of the scrape line (white dotted line) was measured. (TIF) [file pone.0079350.s002.tif]

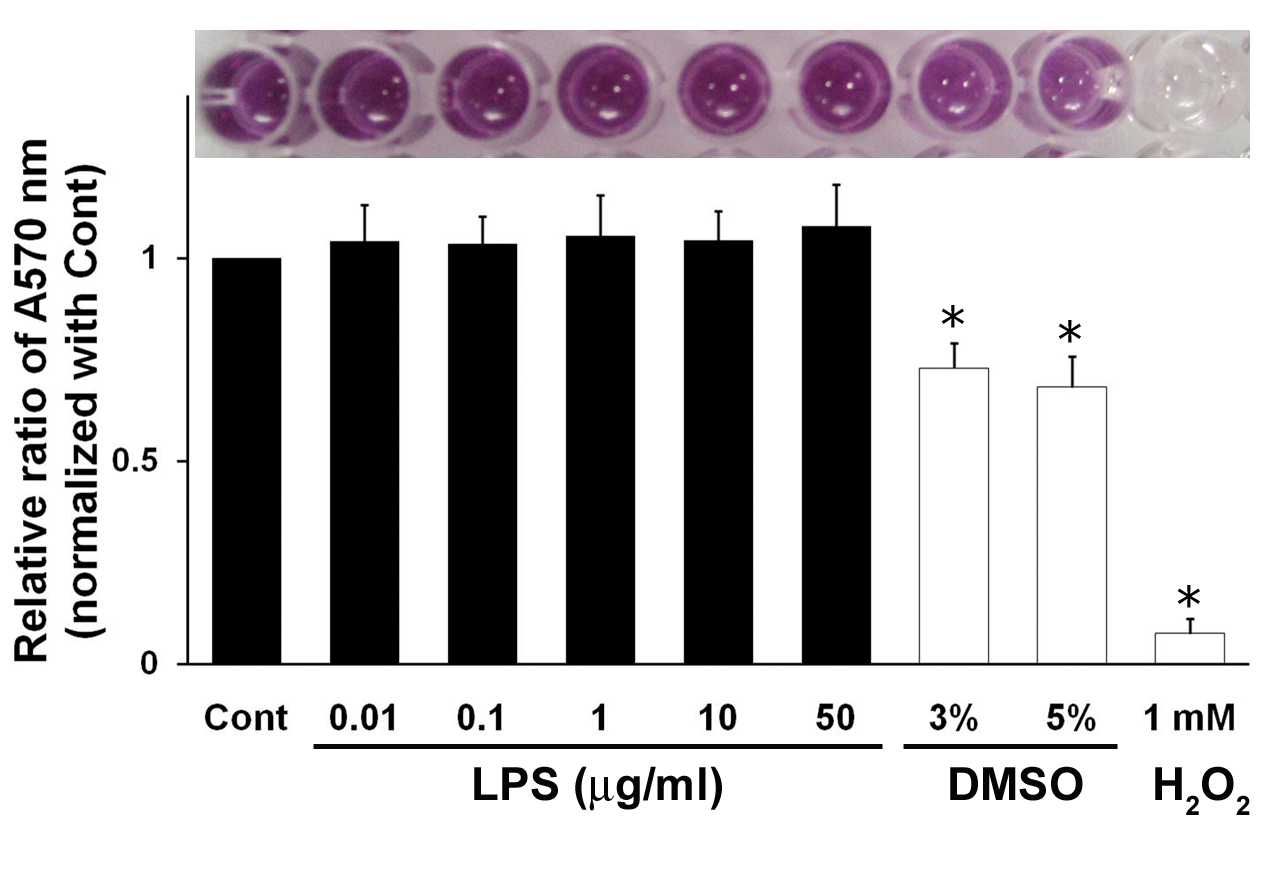

Supplement: Figure S3 — Dose-dependent effect of LPS on astrocyte viability. Control astrocytes (Cont) or astrocytes treated for 24 h with 0.01, 0.1, 1, 10 or 50 µg/ml LPS were subjected to the MTT assay. Cells were also exposed to 3% or 5% DMSO or 1 mM H2O2 as positive control. The upper panel shows the MTT colorimetric assay. The lower panel shows the quantitative data for the MTT test from 3 independent experiments expressed as A570 nm relative to the control. *p<0.01 compared to the control group. (TIF) [file pone.0079350.s003.tif]
